# Expression and regulation of FRMD6 in mouse DRG neurons and spinal cord after nerve injury

**DOI:** 10.1038/s41598-020-58261-7

**Published:** 2020-02-05

**Authors:** Chuang Lyu, Gong-Wei Lyu, Jan Mulder, Mathias Uhlén, Xue-Hui Cai, Tomas Hökfelt, Tie-Jun Sten Shi

**Affiliations:** 1 State Key Laboratory of Veterinary Biotechnology, Harbin Veterinary Research Institute of Chinese Academy of Agricultural Sciences, Harbin, 150069 PR China; 2 Science for Life Laboratory, Department of Neuroscience, Karolinska Institutet, SE-171 65 Stockholm, Sweden; 3 Department of Neurology, 1st Hospital of Harbin Medical University, Harbin, 150001 PR China; 4 Science for Life Laboratory, KTH – Royal Institute of Technology, Stockholm, SE-171 65 Sweden; 5 Department of Neuroscience, Karolinska Institutet, SE-171 77 Stockholm, Sweden; 6 Department of Biomedicine, University of Bergen, 5009 Bergen, Norway

**Keywords:** Neuroscience, Molecular neuroscience

## Abstract

FRMD6, a member of the group of FERM-domain proteins, is involved both in communication between cells, interactions with extracellular matrix, cellular apoptotic and regenerative mechanisms. FRMD6 was first discovered in the rodent sciatic nerve, and in the present immunohistochemical study we investigated the distribution of FRMD6 in the dorsal root ganglia (DRGs), sciatic nerve and spinal cord following sciatic nerve injury. FRMD6-immunoreactivity was found in the cytoplasm, nucleus or both, and in a majority of DRG neurons. FRMD6-immunoreactivity co-existed with several well-known neuronal markers, including calcitonin gene-related peptide, isolectin B4 and neurofilament 200 in mouse DRGs. After peripheral nerve injury, the FRMD6 mRNA levels and the overall percentage of FRMD6-positive neuron profiles (NPs) were decreased in ipsilateral lumbar DRGs, the latter mainly affecting small size neurons with cytoplasmic localization. Conversely, the proportion of NPs with nuclear FRMD6-immunoreactivity was significantly increased. In the sciatic nerve, FRMD6-immunoreactivity was observed in non-neuronal cells and in axons, and accumulated proximally to a ligation of the nerve. In the spinal cord FRMD6-immunoreactivity was detected in neurons in both dorsal and ventral horns, and was upregulated in ipsilateral dorsal horn after peripheral nerve axotomy. Our results demonstrate that FRMD6 is strictly regulated by peripheral nerve injury at the spinal level.

## Introduction

FRMD (protein 4.1, ezrin, radixin and moesin) 6, also known as Willin, is a member of the 4.1 superfamily and was first identified in the sciatic nerve of rats by northern blot analysis^1^. Recently, the FRMD6 transcripts were detected in mouse lumbar dorsal root ganglia (DRGs) and spinal cord by RNAseq (see Supplementary Table 4)^2^. Human FRMD6 has been shown to be a homolog of Ex in Drosophila, which is a putative tumor suppressor^3,4^. FRMD6/Ex, as an upstream regulator, is a component of multiple upstream molecular complexes in the Salvador/Warts/Hippo (Hippo) signaling pathway network that, mostly via MST1/2 (mammalian STE20-like protein kinase) and LATS1/2 (large tumour suppressor homolog), regulates the activity of YAP (Yes-associated protein) and TAZ (transcriptional co-activator with PDZ-binding motif), two downstream homologous transcriptional co-activators^5–8^. This pathway, originally identified in drosophila^9,10^ and more recently shown to be well conserved in mammalians^11^, regulates cellular proliferation, differentiation, apoptosis and tissue homoeostasis^5,6^.

In human, analysing tissues from healthy and diseased donors by immunohistochemistry, FRMD6-immunoreactivity was observed in cytoplasm, plasma membrane or nucleus of various normal tissues (e.g., intestine) and carcinoma, and sometimes with a mixed intracellular distribution^12^. However, the functional associations between these subcellular localizations and physiopathologic processes are still not elucidated. Moreover, FRMD6 transcripts were detected predominantly in fibroblasts within the perineurium of the sciatic nerve in rat, and in the Schwann cells within the endoneurium^13^. Quantitative RT-PCR confirmed that FRMD6 transcripts were expressed at 10-fold higher levels in fibroblasts as compared to Schwann cells^13^. Ectopic expression of FRMD6 activates the Hippo signaling pathway in sciatic nerve fibroblasts, via increasing MST1/2, LATS1/2 and YAP phosphorylation, facilitating YAP translocation from the nucleus to the cytoplasm^8,13^, ultimately preventing TEAD (TEA domain family member)-mediated transcription^9,11,14^. Overexpression of FRMD6 can suppress cell proliferation through antagonizing YAP activity in the sciatic nerve. Peripheral nerve injury causes proliferation of Schwann cells which direct functional repair^15^. An initial nerve injury might result in wound closure induced by FRMD6, and further downregulation of Ephrin B2 and EGFR expression^13^. Upon accumulation of fibroblasts, the activation of YAP would play a predominant role increasing fibroblast proliferation and inhibiting fibroblast migration and also inducing Ephrin B2 and EGFR expression^16^. The expression of Ephrin B2 in fibroblasts can activate its receptors in Schwann cells. This signaling event leads to an organized directional cell migration by the Schwann cells in a Sox2 dependent manner, and guides axon regrowth. Thus, FRMD6 and the Hippo signaling pathway could contribute to the maintenance of homeostasis in the peripheral nervous system. Interestingly, FRMD6 transcripts are also found to be expressed in human^17^ and mouse^2,18^ sensory ganglion neurons, as detected by RNA-seq. Moreover, during early development, aberrant YAP/TAZ can lead to overexpansion of the dorsal root ganglion (DRG) progenitor population^19^, suggesting a functional role of FRMD6 and/or the Hippo signaling pathway in sensory neurons.

In this study, we investigated the expression and regulation of FRMD6 in DRGs and spinal cord in adult mice after a complete peripheral (sciatic) nerve transection using immunohistochemistry (IHC). Our findings indicated that the expression of FRMD6 protein and mRNA was overall significantly downregulated by peripheral nerve injury in the DRGs, whereas the protein was significantly upregulated in the spinal dorsal horn. These data shed a new light on the relationship between sensory neuronal Hippo signaling pathway and peripheral nerve injury.

## Results

### Expression of FRMD6 in control DRGs

**Figure 1:**
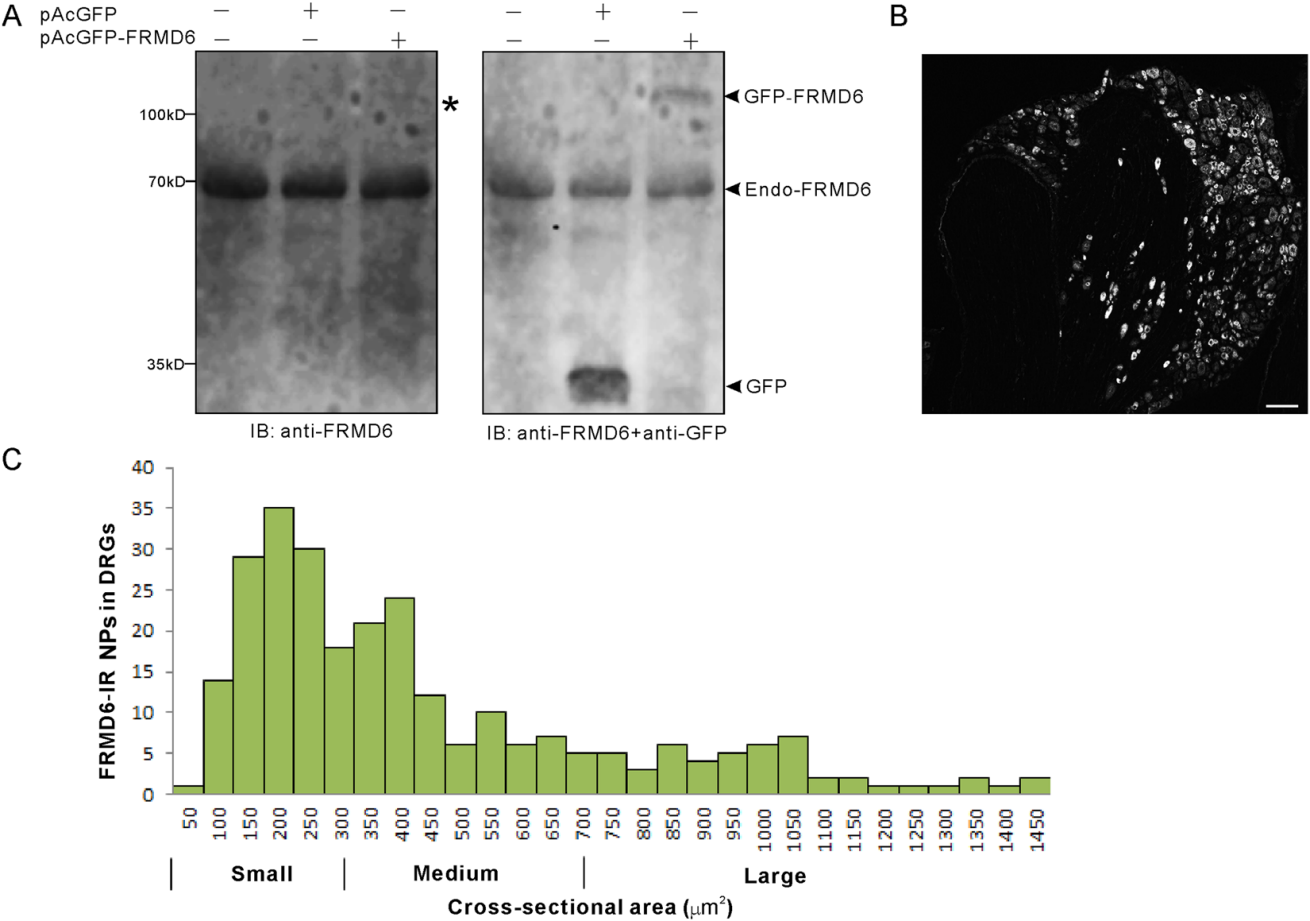
Presence and size distribution of FRMD6-IR neurons in control L4-5 DRGs. (**A**) Identification of specificity of anti-FRMD6 polyclonal antibody used in this study. The NIH 3T3 cells were transfected with pAcGFP or pAcGFP-FRMD6 (1 μg per plasmid), respectively. At 24 hpt, the cell lysates were subjected to Western blot analysis using anti-FRMD6 and followed by anti-GFP antibodies. (**B**) A representative DRG section stained with the anti-FRMD6 polyclonal antibody shows a large number of positive cell bodies. (**C**) Quantitative analysis of FRMD6-IR NPs based on the cross-sectional area. All size categories are represented, small and medium-sized NPs being most common. Scale bar indicates 100 μm (**B**).

The anti-FRMD6 polyclonal antibody was produced using the PrEST antigen which shares 94% homology between human and mouse (https://www.antibodypedia.com/gene/35/FRMD6/antibody/6112/HPA001297). The specificity of this antibody was determined as shown in Fig. 1A. Western blot result showed that anti-FRMD6 polyclonal antibody reacted with green fluorescent protein (GFP) fused human (h) FRMD6 and endogenous mouse (m) FRMD6, but not with GFP, in transfected NIH 3T3 cells, thus indicating a specific reaction with hFRMD6 (Fig. 1A). In addition, the specificity of this antibody was also determined by an RNA interference assay using human cell lines in a previous study^20^.

**Figure 2:**
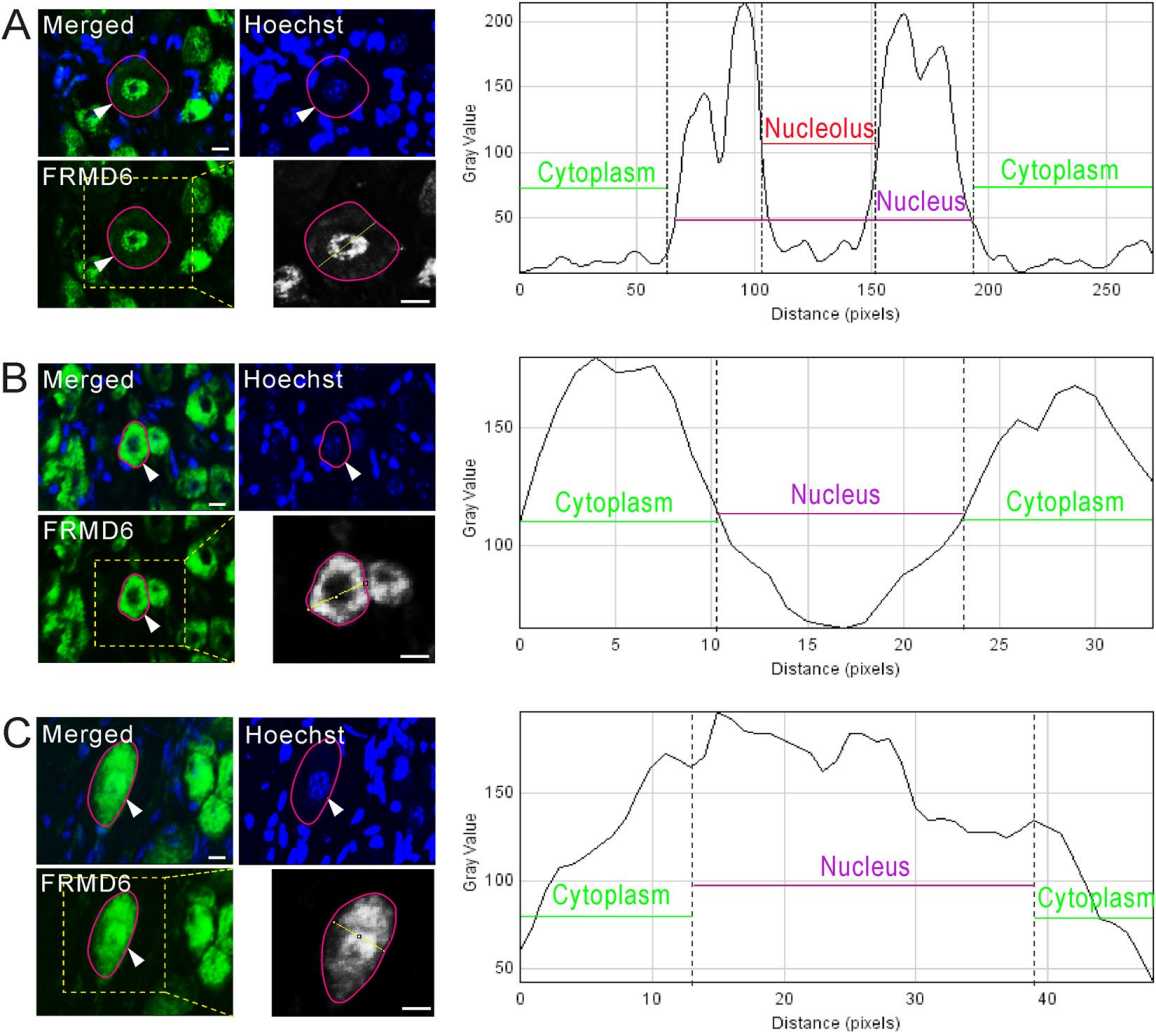
Intracellular localization of FRMD6-immunoreactivity in control L4-5 DRG neurons. (**A–C**) Hoechst is used as a nuclear marker, and the magenta line indicates the border of a NP. Arrowheads indicate the same neuron in A, B and C, respectively. Linear measurement across the whole NP shows intensity level along the line. FRMD6-immunoreactivity is detected only in the nucleus (**A**), only in the cytoplasm (**B**) and both in the nucleus and cytoplasm (**C**). Scale bars indicate 10 μm (**A–C**).

Numerous neurons in mouse DRGs were FRMD6 positive (^+^) (Fig. 1B). Size distribution analysis showed presence in all size categories but with a higher proportion of small and medium-sized neuron profiles (NPs) (Fig. 1C). As shown in Fig. 2, scanning analysis indicated three distinct expression patterns of FRMD6 in DRG neuron somata: nuclear staining (Fig. 2A), cytoplasmic staining (Fig. 2B) and nuclear plus cytoplasmic staining (Fig. 2C). However, the nucleolus always lacked FRMD6-immunoreactivity (Fig. 2A).

### Regulation of FRMD6 in DRGs after axotomy

**Figure 3:**
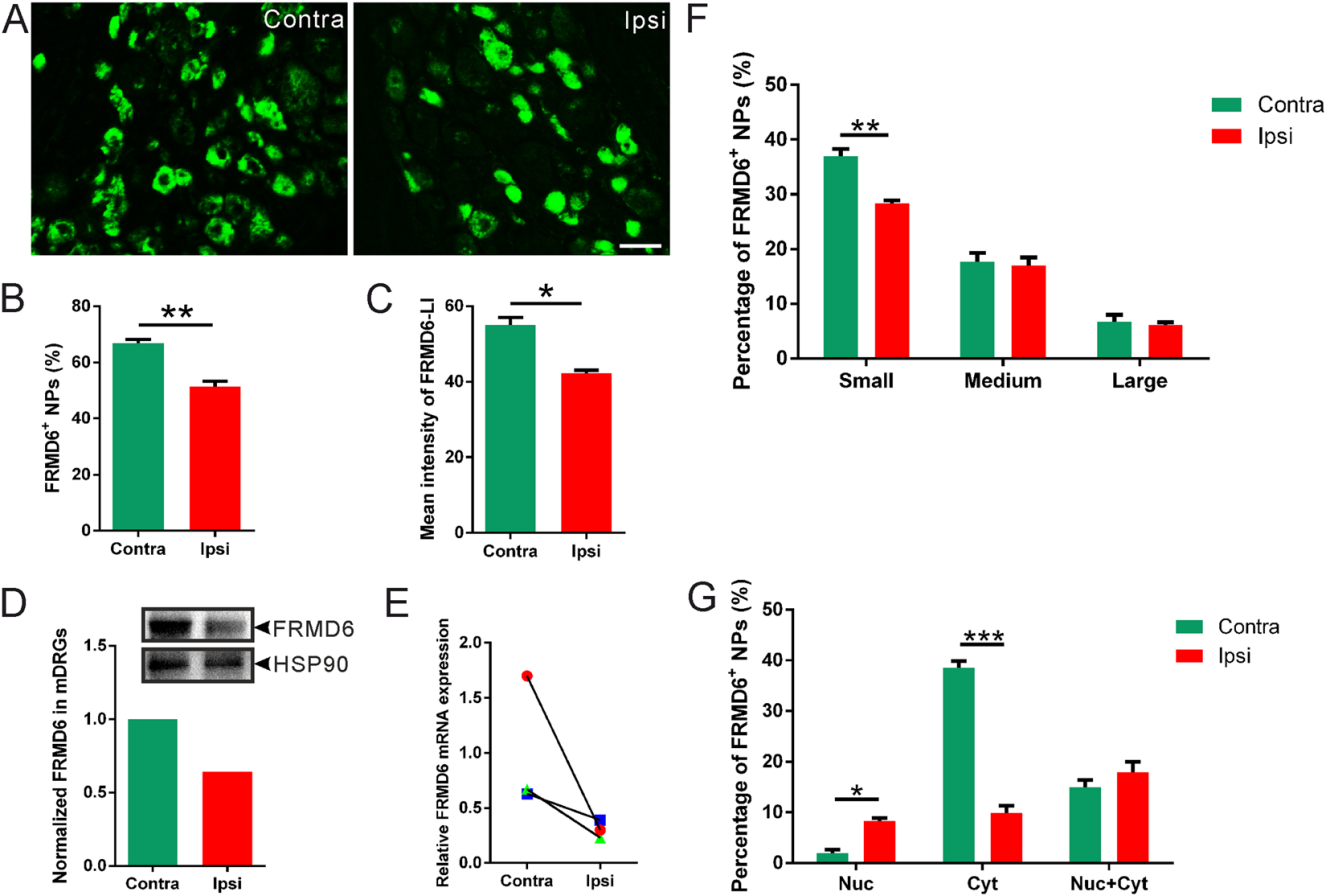
Localization of FRMD6-immunoreactivity in L4-5 DRGs 1 week after axotomy. (**A–C**) There is an ipsilateral downregulation both with regard to percentage of FRMD6^+^ NPs (**B**, n = 6 DRGs per group) and mean fluorescence intensity of FRMD6-immunoreactivity (**C**, n = 6 DRGs per group). (**D,E**) A reduced trend of FRMD6 protein (**D**) and mRNA levels (**E**), in ipsilateral DRGs compared to contralateral ones. (**F**) Percentage of FRMD6^+^ NPs is decreased in the small (<300 μm^2^), but not in the medium-sized (300–700 μm^2^) or large (>700 μm^2^) neuron populations (n = 6 DRGs per group). (**G**) The percentage of NPs with nuclear FRMD6-immunoreactivity is increased and of NPs with cytoplasmic FRMD6-immunoreactivity is decreased versus no difference for those with both nuclear and cytoplasmic FRMD6-immunoreactivity. Scale bar indicates 40 μm (**A**).

Seven days after unilateral sciatic nerve axotomy, a significant downregulation of FRMD6-immunoreactivity was observed in the ipsilateral versus contralateral DRGs (Fig. 3A), both with regard to percentage of FRMD6^+^ NPs (Fig. 3B: 51.4 ± 1.8%, vs. 66.8 ± 1.4%; *p* < 0.01, n = 6 DRGs per group) and mean fluorescence intensity of DRG profiles (Fig. 3C: 42.2 ± 0.7% vs. 55 ± 1.9%; *p* < 0.05, n = 6 DRGs per group). This downregulation was confirmed both by Western blot (Fig. 3D) and qPCR (n = 3 mice per group) (Fig. 3E), indicating reduced expression of FRMD6 after nerve injury. Further analysis showed that this downregulation was predominantly in small NPs, whereas no changes were detected in medium-sized or large NPs (Fig. 3F). Interestingly, the axotomy-induced downregulation was associated with cytoplasmic FRMD6^+^ NPs (Fig. 3G). In addition, there was a significant increase in proportion of nuclear FRMD6^+^ NPs (Fig. 3G). No effect was observed on the population of neurons with nuclear plus cytoplasmic FRMD6^+^ NPs (Fig. 3G).

### Expression of FRMD6 in subpopulations of DRG neurons

**Figure 4:**
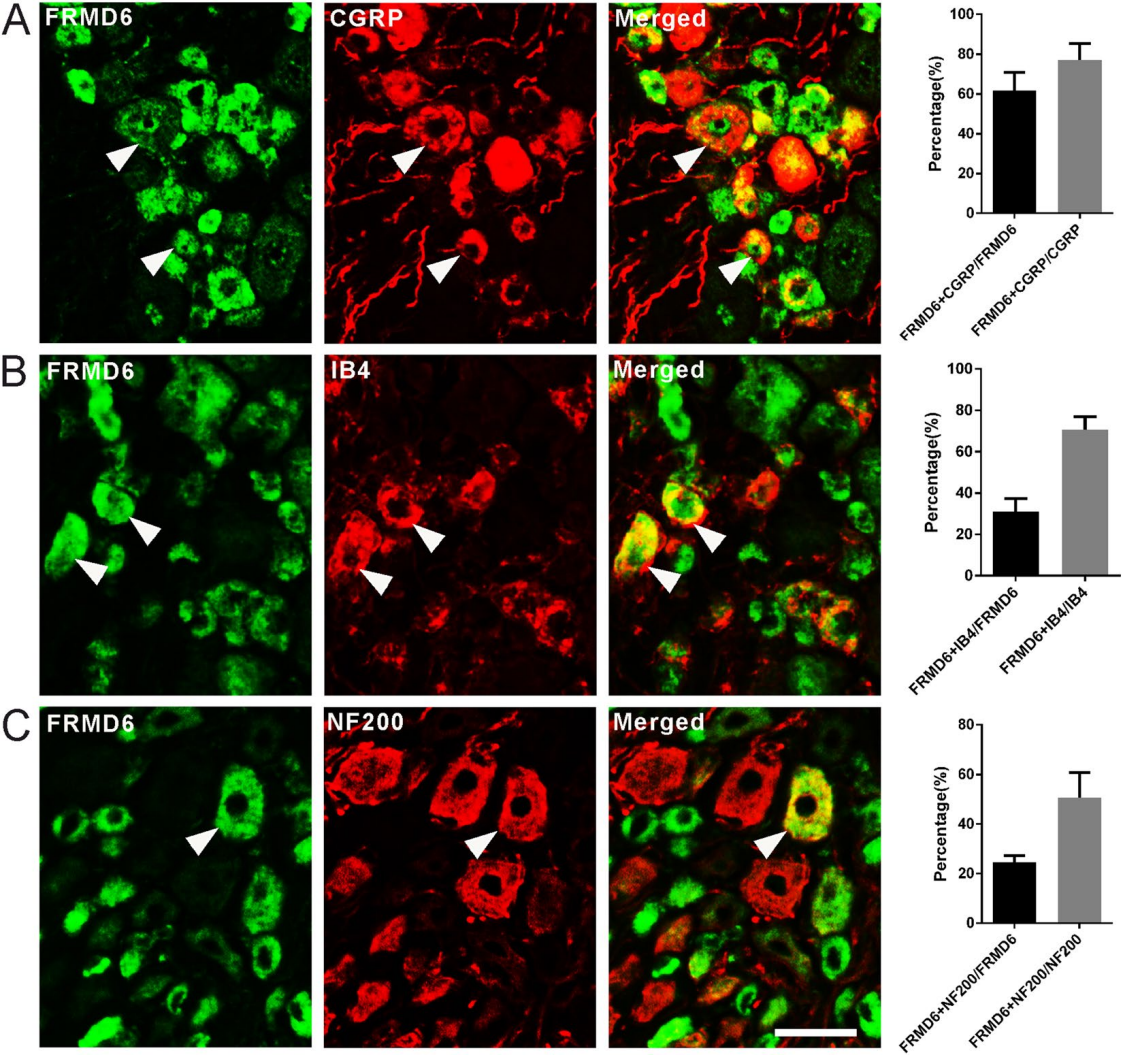
Co-existence of FRMD6-immunoreactivity with three neuronal markers in contralateral L4-5 DRGs. (**A**–**C**) FRMD6 co-exists with CGRP (**A**), IB4 (**B**) and NF200 (**C)**. The highest degree of co-existence is seen for CGRP. Arrowheads indicate neurons with co-existence. Scale bar indicates 40 μm (**A–C**).

Calcitonin gene-related peptide (CGRP), isolectin B4 (IB4) and neurofilament 200 (NF200) were employed as phenotypic markers to differentiate small unmyelinated peptidergic, small unmyelinated non-peptidergic and medium–large myelinated neurons, respectively, and peripheral small unmyelinated (C-fiber) neurons which are known as nociceptors^21^. In contralateral DRGs, 61.6 ± 9.2%, 30.9 ± 6.3% and 24.7 ± 2.6% of FRMD6^+^ NPs expressed CGRP (Fig. 4A), IB4 (Fig. 4B) and NF200 (Fig. 4C), respectively. Conversely, 77.1 ± 8.2%, 70.6 ± 6.3% and 50.7 ± 10.1% of CGRP^+^, IB4^+^ and NF200^+^ NPs expressed FRMD6-immunoreactivity, respectively (Fig. 4). In ipsilateral DRGs, 59.8 ± 9.2%, 25.7 ± 0.7% and 40.3 ± 7.6% of FRMD6^+^ NPs expressed CGRP, IB4 and NF200, respectively. Conversely, 91.5 ± 4.7%, 60.2 ± 3.3% and 46.4 ± 6.3% of CGRP^+^, IB4^+^ and NF200^+^ NPs expressed FRMD6-immunoreactivity in ipsilateral DRGs, respectively (Fig. S1).

**Figure 5:**
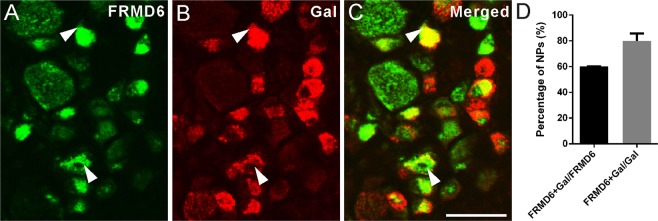
FRMD6 coexists with galanin (Gal) in ipsilateral L4-5 DRGs one week after axotomy. FRMD6 (**A**) co-exists with Gal (**B**), as shown in (**C**). (**D**) Co-existence quantification of FRMD6 and Gal. Many FRMD6^+^ neurons express Gal-immunoreactivity. Scale bar indicates 40 μm (**A**–**C**).

We also analyzed expression of the 29-amino acid neuropeptide galanin^22^ which has been shown to be strongly upregulated in DRG neurons after peripheral nerve injury^23^. Thus, 59.9 ± 0.3% of FRMD6^+^ NPs expressed galanin in ipsilateral DRGs. Conversely, FRMD6-immunoreactivity was observed in 79.8 ± 5.9% of the galanin^+^ NPs in ipsilateral DRGs (Fig. 5A–D).

### FRMD6 is axonally transported in the sciatic nerve

**Figure 6:**
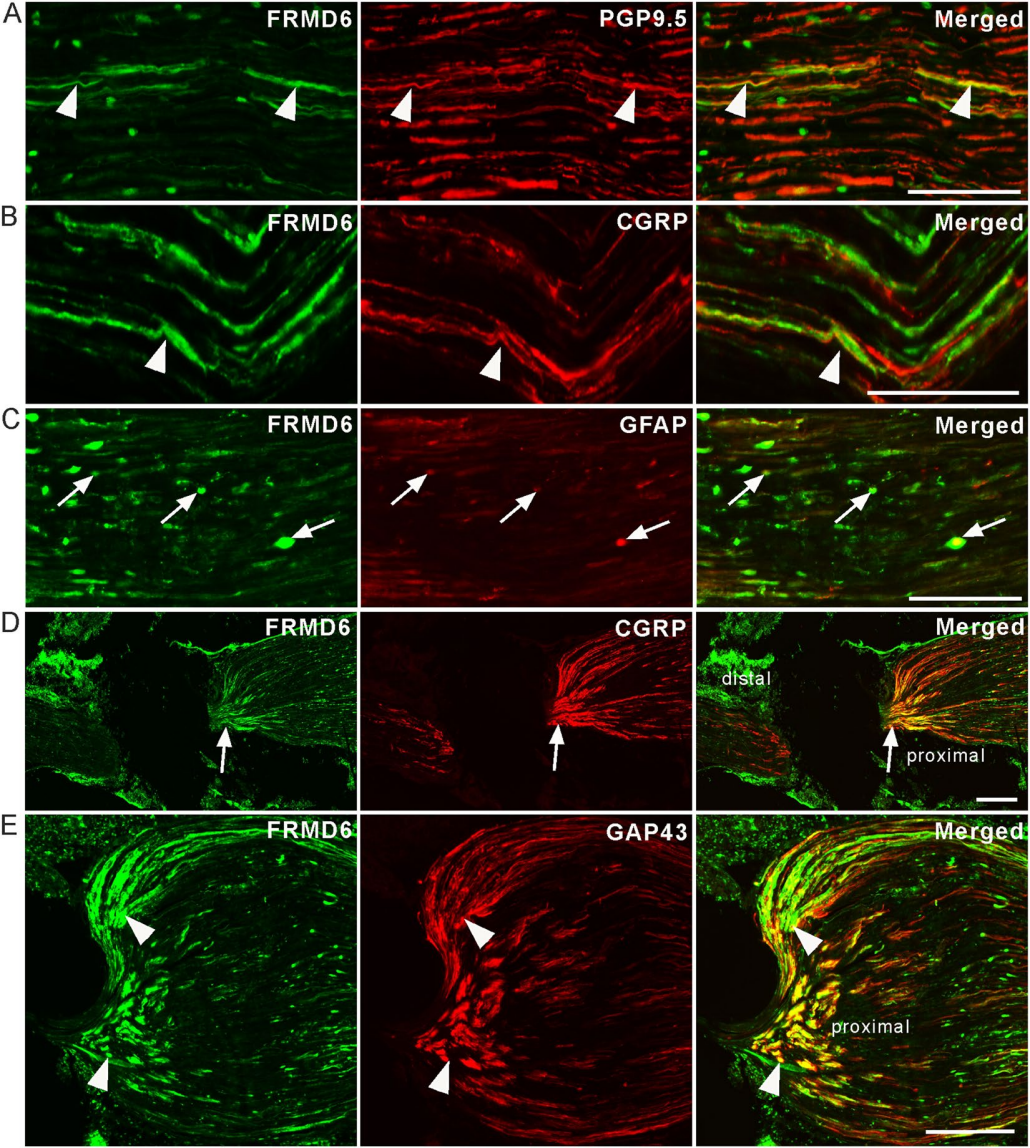
Localization of FRMD6 in the sciatic nerve, normally (**A**–**C**) and 10 hours after nerve ligation (**D,E**). (**A**) FRMD6-immunoreactivity is seen in PGP9.5^+^ axons (arrowheads). (**B**) FRMD6-immunoreactivity is observed in some CGRP^+^ axons (arrowheads). (**C**) FRMD6 co-localizes with the Schwann cell marker GFAP (arrows). (**D**) Ligation causes a strong accumulation of FRMD6-immunoreactivity on the proximal side of the nerve, in parallel with CGRP-immunoreactivity; merged micrograph shows overlap and co-existence. (**E**) At high magnification FRMD6-immunoreactivity has a high degree of co-existence with GAP43-immunoreactivity proximal to the ligation (arrowheads). Scale bars indicate 50 μm (**B**), 100 μm (**A,C**), 200 μm (**D,E**).

FRMD6-immunoreactivity was observed in the sciatic nerve of mice, and many fibers were labeled with the axonal marker PGP9.5 (Fig. 6A). Some FRMD6^+^ fibers were CGRP^+^, in agreement with evidence that the FRMD6 and CGRP coexist in the neuronal cell bodies in DRGs (Fig. 6B). In addition, FRMD6-immunoreactivity was observed in widely distributed, small, dot-like structures also being GFAP-immunoreactive (-IR), presumably representing Schwann cells (Fig. 6C). Ten hours after sciatic nerve ligation, a strong accumulation of FRMD6-immunoreactivity was seen in the proximal stump (Fig. 6D,E). Many of the abundant FRMD6^+^ fibers proximal to the ligation site were CGRP^+^ (Fig. 6D), and even more GAP43^+^ (Fig. 6E). The latter molecule is a biomarker for growing axons in development and regeneration^24,25^.

### FRMD6 distribution in the spinal cord after unilateral axotomy

**Figure 7:**
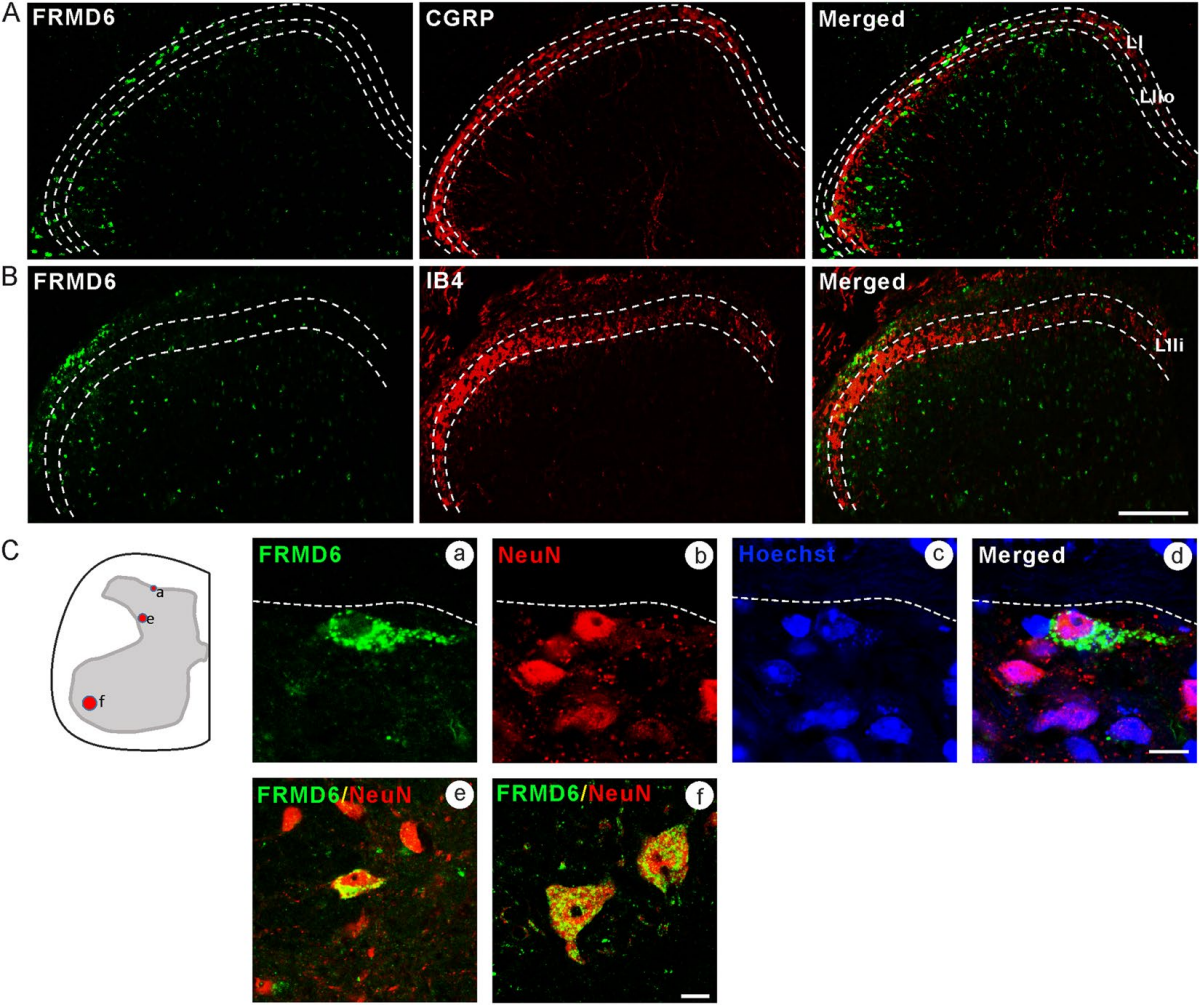
Localization of FRMD6-immunoreactivity in control L4-5 spinal cord. (**A**) Partial overlap of FRMD6- with CGRP-immunoreactivity, as shown with double-staining. CGRP antiserum labels lamina I (LI) and outer lamina II (LIIo) layers. (**B**) Overlap of FRMD6-immunoreactivity with IB4-binding, as shown with double-staining. IB4 labels inner lamina II (IIi) layer. Note many FRMD6^+^ cells in deeper layers. (**C**) Co-localization of FRMD6 with NeuN in local neurons in spinal dorsal horn (a–e) and motor neurons in ventral horn (f). Scale bar indicates 100 μm (**A,B**), 15 μm (C; f) and 10 μm (**C**; a–e).

**Figure 8:**
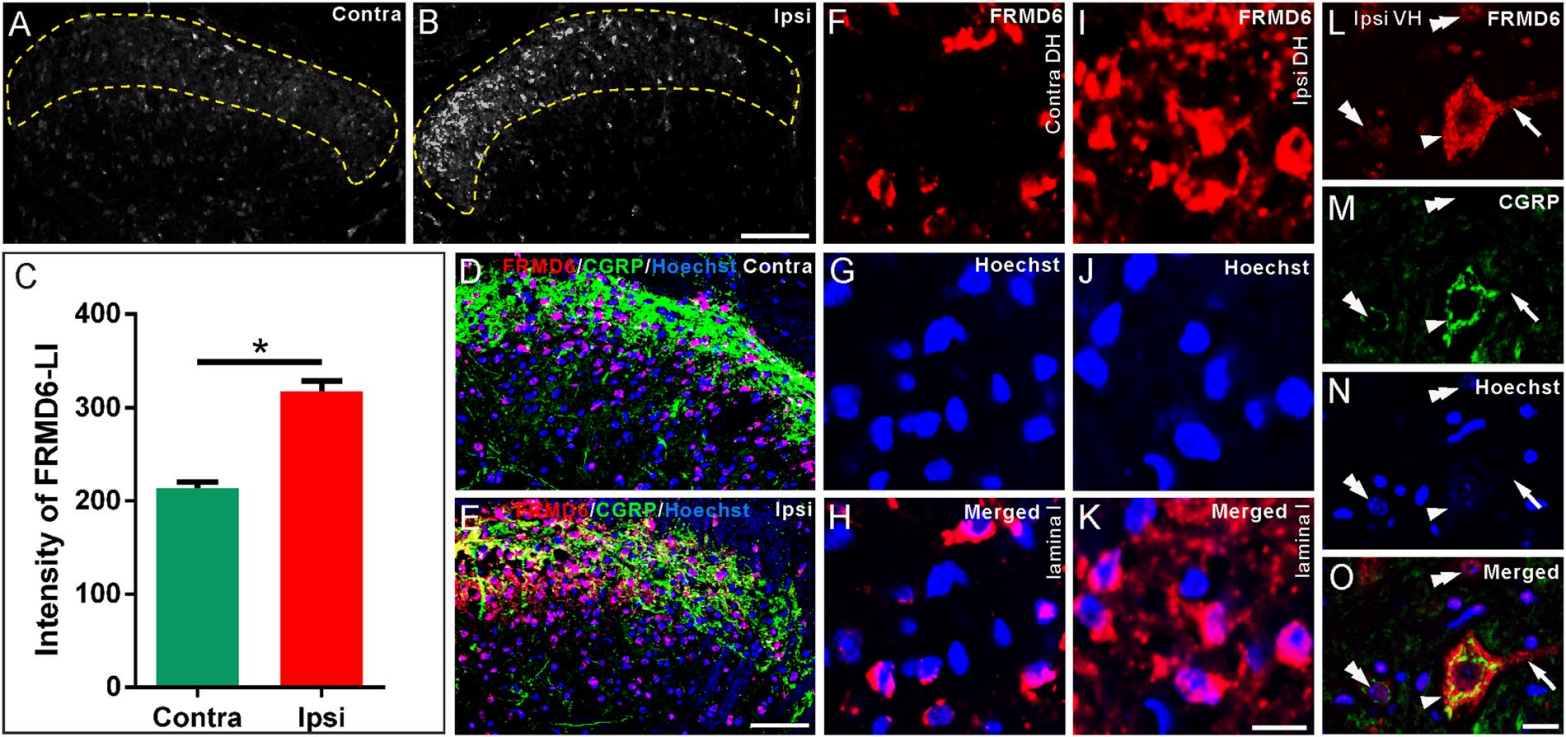
Localization of FRMD6-immunoreactivity in L4-5 spinal cord one week after sciatic nerve axotomy. (**A,B**) FRMD6-immunoreactivity is increased in the ipsilateral compared to the contralateral dorsal horn (DH). (**C**) Mean intensity of FRMD6-immunoreactivity in the superficial layers (outlined by yellow dashed lines) is significantly increased on the ipsilateral side (n = 3 spinal cord). (**D,E**) Triple labeling for FRMD6, CGRP and Hoechst in the contralateral (**D**) and ipsilateral DH (**E**). Note increase in FRMD6-immunoreactivity and decrease in CGRP-immunoreactivity. (**F–K**) High magnification micrographs show FRMD6-immunoreactivity is increased in ipsilateral (**I**,**K**) than contralateral side (**F**,**H**). (**L–O**) After triple staining (**O**) for FRMD6 (**L**), CGRP (**M**) and Hoechst (**N**), FRMD6-immunoreactivity is seen in a cell body (arrowheads) and processes (arrows) of a CGRP^+^ motor neuron in the ipsilateral ventral horn (VH). Weak nuclear labeling for FRMD6 is seen in CGRP^+^ (left double arrowheads) or CGRP-negative (right top double arrowheads) neurons. Scale bars indicate 100 μm (**A,B**), 50 μm (**D,E**) and 10 μm (**F–O**).

FRMD6-IR NPs were observed in the control lumbar (L) 4–5 spinal cord segments, including dorsal horn (DH) and ventral horn (VH) (Figs. 7 and 8). Based on *in situ* hybridization, a similar overall distribution of FRMD6 mRNA at the lumbar level in adult C57BL/6 J mice has been reported in the ALLEN BRAIN ATLAS (Fig. S2, modified from original data downloaded from: http://mousespinal.brain-map.org/imageseries/detail/100033091.html). The superficial layers (Laminae I-IIi) of spinal dorsal horn were labeled with CGRP-immunoreactivity and IB4 binding which indicated nerve terminals from DRG neurons. We did not observe an apparent nerve terminal-like distribution of FRMD6-LI in these regions (Fig. 7A,B). The FRMD6-immunoreactivity was found in the cytoplasm of local neurons in the spinal dorsal horn using NeuN as a marker (Fig. 7C; a-e). In addition, both nuclear and cytoplasmic distribution was observed in the ventral horn NPs (Fig. 7C; f).

After axotomy, there was a distinct increase in FRMD6-immunoreactivity in the ipsilateral dorsal horn, mainly in superficial layers (Fig. 8A–C, and F vs. I). In the spinal ventral horn, FRMD6-immunoreactivity was mainly found in the cytoplasm of large neurons and coexisted with CGRP-immunoreactivity, supporting their motor neuron character, here shown on the ipsilateral side (Fig. 8L–O). Note the different cytoplasmic localization of FRMD6- (Fig. 8L,O) and CGRP-immunoreactivity (Fig. 8M,O). The former was either spread diffusely throughout the cytoplasm and into processes with a low nuclear content, or apparently had only a nuclear localization (Fig. 8L–O), whereas CGRP was stored in perinuclear patches and in neuronal processes (Fig. 8L–O). There were also examples of nuclear FRMD6-immunoreactivity without CGRP-immunoreactivity and with a very weak or discrete CGRP-immunoreactivity (Fig. 8L–O).

## Discussion

Expression of FRMD6 mRNA has previously been reported in fibroblasts and Schwann cells in the rat sciatic nerve using *in situ* hybridization^1,13^. In the present immunohistochemical study, we in addition detected FRMD6-immunoreactivity, i.e. FRMD6 protein in cell bodies of various sizes in DRGs and in their axons in the sciatic nerve, and confirmed its presence in Schwann cells by co-labeling with an antibody against GFAP, a nonmyelin-forming Schwann cell marker^26,27^. Moreover, the expression and cellular distribution of FRMD6 in DRG and spinal cord neurons were altered by peripheral nerve injury. FRMD6-immunoreactivity was also found in neurons of L4-5 lumbar spinal cord, including motor neurons. These findings suggest that FRMD6 is probably of functional significance in all categories of DRG neuron, i.e. is likely involved in many sensory modalities and in motor behavior.

The presence and functional role of FRMD6 in adult primary sensory neurons have until now not been explored. However, RNA-seq analyses have demonstrated presence of FRMD6 transcript in human and mouse DRGs, respectively^17,28^. Thus, one aim was to clarify in some detail the localization of the FRMD6 in sensory neurons of DRG and in spinal cord. In agreement with previous findings on other systems^12^, we observed both cytoplasmic and nuclear localization, whereas no direct plasma membrane association was reported in epithelial cells^29^. In the DRG neurons, FRMD6-immunoreactivity was mostly present in the small and medium-sized neuronal populations, many of which are nociceptors, in addition to some large neurons.

A second aim was to understand a possible pathomechanistic role of FRMD6 after complete sciatic nerve axotomy. Our quantitative analysis showed that this procedure caused distinct changes in FRMD6 expression based on subcellular localization and neuronal size. Both FRMD6 protein and transcript levels decreased ipsilaterally, but only in the small-size neuron population, containing the nociceptors. In parallel, there was an ipsilateral increase in the proportion of nuclear FRMD6^+^ NPs, thus probably suggesting a translocation.

Functionally, FRMD6 is known as an upstream member involved in the regulation of Hippo signaling pathway with YAP as a final executor. YAP controls, among others, cellular proliferation, e.g. of the DRG progenitor population, differentiation and regeneration^5,9,30–33^. It has been reported that overexpression of FRMD6 can antagonize YAP activity^8,14^, mainly via an action in the cytoplasm^34^. Conversely, decreased levels of FRMD6 lead to translocation of YAP from cytoplasm to nucleus, inducing transcriptional activation, which can result in opposite effects, including increased cell proliferation^7,14^. It has been shown that a chronic sciatic nerve construction induces nuclear accumulation of YAP/TAZ in the spinal dorsal horn^35^. Moreover, spinal inhibition or knockdown of YAP/TAZ significantly suppresses mechanical allodynia^35^. Thus, regulation of YAP/TAZ is a core mechanism underlying neuropathic pain. Given the important role played by Hippo signaling pathway in regulating YAP/TAZ activity, these authors also analyzed the function of FRMD6, an upstream component, in YAP-induced neuropathic pain. Interestingly, knockdown of FRMD6 led to a nuclear accumulation of YAP through a reduction of a series of phosphorylation events, including p-MST1/2, p-LAST1 and finally p-YAP, in the rat spinal dorsal horn. Moreover, an intrathecal FRMD6 siRNA application induced a long-lasting mechanical allodynia, indicating an analgesic role of FRMD6 in spinal cord^35^. The present results suggest that the lower levels of cytoplasmic FRMD6 in injured DRG neurons may attenuate its inhibitory action on the Hippo signaling pathway, that is nuclear YAP will be upregulated and could then promote neuropathic pain.

It is well known that peripheral nerve injury can cause varying degrees of neuronal death in DRGs^36–38^. In fact, in mice a significant loss of DRG neurons occurs after 2 weeks, even more remarkable after one month^39^. The Hippo signaling pathway has been shown to regulate cell proliferation as well as apoptosis to control organ size in a variety of animals^9,40^. The activation of YAP (nuclear translocation) can promote cell proliferation and inhibit apoptosis through regulation of specific gene transcriptions. Thus, the downregulation of cytoplasmic FRMD6 could result in an activation of YAP nuclear translocation through modulating Hippo signaling pathway, and thereby influence neuronal survival in DRGs after nerve injury. Despite this, the function of increased expression (or possible translocation) of nuclear FRMD6 is still need to be clarified. It would be interesting to explore to what extent altered FRMD6 expression and distribution correlate with neurodegenerative processes.

We have extensively studied the neuropeptide galanin^22^, another molecule that is important in relation to injury to the sciatic nerve^41^. Here we detected a high degree of coexistence of galanin- and FRMD6-immunoreactivity in DRGs after peripheral axotomy. This is in agreement with the strong upregulation of galanin in DRG neurons after peripheral nerve injury^23^. Galanin has been shown to have trophic effects supporting regeneration via its Gal_2_-R receptor^42^ as well as to influence (neuropathic) pain signaling^41^. Therefore, it is possible that FRMD6 and galanin, independently and by completely different mechanisms, cooperate to counteract the consequences of nerve injury, that is by promoting regeneration and alleviate pain. In fact, galanin is just one of hundreds of molecules in DRGs that are altered after nerve injury^43,44^.

In the intact sciatic nerve FRMD6-immunoreactivity could be detected both in nerve fibers (all PGP9.5^+^ and some CGRP^+^) and non-neuronal cells, some of which were GFAP^+^, thus likely Schwann cells, the latter in agreement with data on transcripts by Moleirinho *et al*.^13^. Ligation of the sciatic nerve shows a marked accumulation of FRMD6-immunoreactivity on the proximal side and thus evidencing anterograde axonal transport. No attempts were made to visualize the protein in nerve endings in the skin. At the ligation site FRMD6-immunoreactivity strongly co-accumulated with GAP43-immunoreactivity, a marker for nerve regeneration, in line with previous studies linking FRMD6 and Hippo signaling pathway to regenerative mechanisms.

Our results show a wide distribution of the FRMD6 protein in the mouse spinal cord, in agreement with the distribution of its transcript shown in the ALLEN BRAIN ATLAS^45–51^. The increase of FRMD6-immunoreactivity in laminae I-II seven days after sciatic nerve axotomy likely occurs in local neurons; it is less likely to be associated with primary afferents, also because there is a downregulation in cytoplasmic FRMD6 and its mRNA in DRG neuron cell bodies after axotomy.

We also demonstrate FRMD6-immunoreactivity CGRP^+^ motor neurons and found that FRMD6-immunoreactivity is colocalized with ATF3-immunoreactivity in ipsilateral, lesioned motor neurons (Fig. S3). This is in agreement with previous finding of an ipsilateral ATF3^+^ signal at mRNA and protein levels in the nucleus of motor neurons after exposure to stresses including nerve injury^52^. ATF3 can repress transcription by binding to DNA sites as a homodimer or heterodimer with Jun proteins^53–55^, suggesting a possible combination of FRMD6 with nuclear DNA or other stress-induced molecules.

## Methods

### Animals and cell cultures

Animal experiments were performed on 12–14 weeks old male C57BL/6 J Bommince mice (A/S Bomholtgaard, Ry, Denmark) weighing 25–30 g. All animals were kept under standard conditions on a 12-hour day/night cycle with free access to food and water.

NIH 3T3 and HEK293T cells were cultured in Dulbecco’s Modified Eagle’s Medium (DMEM; GIBCO, US) supplemented with 10% fetal bovine serum (FBS; GIBCO, US) and 1% penicillin-streptomycin, and kept at 37 °C in an atmosphere of humidified 5% CO_2_.

### Ethics

This study has been approved by the local Ethical Committee for animal experiments (Stockholms Norra Djursförsöksetiska Nämnd; N150/11). The evaluation of autotomy behavior development in hind paws of WT C57 BL/6J mice has been reported in our recent paper^56^ that is around 29% mice developed autotomy with a score around 1 on day 7 followed axotomy. From the ethical standpoint, we had to sacrifice the animals when they developed severe autotomy behavior. The criterion was referred to a previous study^57^. Efforts were made to minimize the number and discomfort of animals throughout the study. All experiments were performed in accordance with relevant guidelines and regulations.

### Plasmid construction and transient transfection

The hFRMD6 open reading frame was polymerase chain reaction (PCR) amplified from cDNA of HEK293T cells with forward primer: 5′-GCAGATCTATGAACAAATTGAATTTTCATAAC-3′ and reverse primer 5′- GCGGTACCTTACACAACAAACTCTGGAAC-3′. The PCR was performed using KOD FX Neo DNA polymerase (TOYOBO, Japan) according to the manufacturer’s instructions at a final 50 μL reaction volume containing 2 × buffer (25 μL), forward primer (2.5 μL; 10 μM), reverse primer (2.5 μL; 10 μM), KOD FX Neo (1.25 μL; 1 unit/μL), dNTP (7.5 μL; 2 mM each), cDNA (~200 ng) and ddH_2_O. The PCR product was cloned into vector pAcGFP-C1 (Clontech) at *Bgl* II and *Kpn* I sites. The recombinant plasmid pAcGFP-FRMD6 was verified by sequencing.

Transient transfection was performed by using X-tremeGENE HP DNA transfection reagent (Roche, US) according to the manufacturer’s instructions. Briefly, 1 μg plasmid (pAcGFP-C1 or pAcGFP-FRMD6), 2 μl transfection reagent and 100 μl DMEM were gently mixed by pipetting, and incubated for 20 min at room temperature (RT). The mixture was evenly dropped onto NIH 3T3 cells.

### Surgeries

Studies have shown that the full length of sciatic nerve is around 20 mm in adult male C57BL/6 mouse^60^. In present study a unilateral, complete-sciatic nerve transection (axotomy) was performed as described in our previous study^39^. Briefly, mice were anesthetized with sodium pentobarbital (Mebumal, Apoteket, Stockholm, Sweden) (10 mg/kg, i.p.) and the left sciatic nerve was transected at ‘mid-thigh’ level (around 10 mm distal to the DRG). A 5-mm portion of the nerve was resected, and the proximal end was ligated to prevent regeneration. The animals were allowed to survive for 7 days after surgery. Unilateral sciatic nerve ligation was performed as described previously^58,59^. The procedure of transport studies was carried on a previously published but modified animal model. A mid-thigh ligation was made as followed: a skin incision (5 mm) was made vertically along the mid-thigh level. The muscles of the posterior thigh were split distally to expose the sciatic nerve. The sciatic nerve was carefully isolated by forceps until a point where the nerve separated into two branches, i.e. the sural nerve and tibial nerve. From this point the ligation (black silk suture, 6–0, sterile, Art. No. 14719, AgnThos) was introduced at a 10 mm place along the sciatic nerve. The skin was then closed with the silk suture. After the surgery, the animals were returned to their original cages. Ten hours after surgery animals were deeply anesthetized and perfused. Throughout this manuscript, the tissues from untreated (also known as normal) mice were used as control. In addition, the tissues from the uninjured (contralateral) side were compared with those from injured (ipsilateral) side.

### Immunohistochemistry

Animals were deeply anesthetized with Mebumal (50 mg/kg, i.p.), transcardially perfused with 20 ml warm saline (0.9%, 37 °C), followed by 20 ml of a mixture of 4% paraformaldehyde and 0.4% picric acid in 0.16 M phosphate buffer (pH 7.2, 37 °C), and then by 50 ml of the same, but ice-cold fixative. The L4–5 DRGs and L4-5 segment of spinal cord and sciatic nerve were dissected, and post-fixed in the same fixative for 90 min at 4 °C. Specimens were subsequently stored at 4 °C for 2 days in 10% sucrose in phosphate buffered saline (PBS, 0.1 M, pH 7.4) containing 0.01% sodium azide (Sigma) and 0.02% bacitracin (Sigma) as preservatives.

Tissues were embedded in OCT compound (Tissue Tek, Miles Laboratories, Sakura, Leiden, Netherland), sectioned in a cryostat (Microm, Heidelberg, Germany) at 12 μm (DRGs) or 20 μm (sciatic nerve and spinal cord) thickness and mounted onto slides (SuperFrost Plus, Thermo, Waltham, MA). Sections were dried at RT for 30 min and rinsed with PBS for 10 min. Sections were incubated with rabbit anti-FRMD6 polyclonal antibody diluted in PBS containing 1% (w/v) bovine serum albumin (BSA) and 0.03% Triton X-100 (Sigma) at 4 °C overnight. Immunoreactivity was visualized using the tyramide signal amplification system (TSA Plus; NEN Life Science Products, Boston, MA) as described previously^61^. For double-staining assays, indirect immunohistochemistry was performed on FRMD6-stained sections, and the antibodies and optimal working concentrations are shown in Table 1. To detect neurons which bind with isolectin B4 (IB4), the sections were incubated with IB4 from Griffonia simplicifolia I (GSA I; IB4; 2.5 g/ml; Vector Laboratories, Burlingame, CA) followed by incubation with a goat anti-GSA I antiserum (1:2,000; Vector Laboratories)^62^. The information for primary antibodies is shown in Table 1.

**Table 1:** Primary antibodies.

Antibody	Host	Experiment	Dilution	Supplier/Catalogue #
FRMD6	Rabbit	IHC (TSA)	0.25 μg/ml	Sigma/HPA001297
		IHC (Coons)	1 μg/ml	Sigma/HPA001297
		WB	0.4 μg/ml	Sigma/HPA001297
CGRP	Rabbit	IHC (Coons)	1:2,000	S.I. Grigis
NF200	Mouse	IHC (Coons)	1:400	Sigma/N0142
Galanin	Rabbit	IHC (Coons)	1:800	E. Theodorsson
GFAP	Mouse	IHC (Coons)	1:400	Santa Cruz Biotechnology/sc-33673
GAP43	Rabbit	IHC (Coons)	1 μg/ml	Atlas Antibodies AB/HPA015600
PGP9.5	Rabbit	IHC (Coons)	1:1,600	Ultra Clone
NeuN	Mouse	IHC (Coons)	1:400	MILLIPORE/MAB377
HSP90	Rabbit	WB	1:10,000	Cell Signaling Technology/#4874
GFP	Mouse	WB	1:20,000	66002-1-lg/Proteintech

### Western blot

At 24 hour post transfection (hpt), the transfected HIH 3T3 cells were harvested and lysed in NP40-lysis buffer (Beyotime Biotechnology, China) containing 1 mM PMSF and complete protease inhibitor cocktail (Sigma-Aldrich) for 1 h on ice.

On day 7 after axotomy, four mice were sacrificed, and both contra- and ipsilateral L4–5 DRGs were removed and immediately snap frozen on dry ice. Contralateral and ipsilateral DRGs were separately pooled and placed in lysis buffer containing protease inhibitor (P8340; Sigma), and then properly sonicated at 4 °C.

All lysates were centrifuged at 12,000 rpm for 30 min at 4 °C. The supernatant was collected for Western blot analysis. Tissue protein concentration was measured by Bradford’s Assay (Bio-Rad, Hercules, CA). Laemmeli sample buffer containing around 20 μg of protein was loaded in each lane and separated on 12% SDS-PAGE gel, then transferred to polyvinylidene difluoride membrane (Millipore, Hemel, Hempstead, UK). The membrane was blocked with 5% non-fat dry milk (BIO-RAD, Hercules, CA) in tris-buffered saline (TBS) with 0.1% Tween-20 (TBST) for 1 h at RT and incubated overnight at 4 °C with anti-FRMD6 polyclonal antibody diluted in PBS containing 5% BSA and 0.03% Triton X-100 (Sigma). The membrane was rinsed in TBST three times, and then incubated with HRP-conjugated secondary antibody (1:10,000; DAKO, Glostrup, Denmark) for 1 h at RT, and exposed to ECL solution for 5 min (BIO-RAD).

To detect the endogenous FRMD6 and overexpressed GFP and GFP-hFRMD6 in NIH 3T3 cells, the membrane was incubated with anti-FRMD6 polyclonal antibody, followed by incubation of an anti-GFP monoclonal antibody.

To detect the FRMD6 in contral- and ipsilateral mouse DRGs, the membrane was incubated with anti-FRMD6 polyclonal antibody for detection of FRMD6, and then stripped and re-probed for detection of HSP90 (1:10,000; Cell Signaling Technology, Danvers, UK) as loading control. The intact membranes in Western blot assays were shown in Fig. S4.

### Real-time quantitative PCR

Total RNA was isolated from contralateral and ipsilateral L4–5 DRGs of mice (n = 3 mice) 7 days after axotomy. The quantity and quality of RNA was determined with Nano Drop (Saveen & Werner AB, Malmo, Sweden). For qPCR amplification, 17 ng of RNA was retrotranscribed using iScript Select cDNA Synthesis Kit (BIO-RAD, US). The cDNA samples were subjected to 40 cycles using SYBR Green PCR Master Mix Kit (Applied Biosystems, US). Triple loads of cDNA were set for each sample. The following primers were used for FRMD6 (forward, 5′-CACAGAGGTTGGTCCGAAAT-3′; reverse, 5′-CTGGGAAGGTCATGGAAGAA-3′), and for GAPDH (forward, 5′- CTCTCTGCTCCTCCCTGTTCT-3′; reverse, 5′-TCCGTTCACAC CGACCTT-3′). The PCR procedure was: 50 °C for 2 min, 95 °C for 10 min, followed by 40 consecutive cycles of 95 °C for 15 sec and 60 °C for 1 min. The amplification was performed in 96-well PCR plates in an automated fluorimeter (ABI Prism 7000 Sequence Detector System; Applied Biosystems, Palo Alto, CA). The FRMD6 mRNA levels were expressed as a ratio to GAPDH mRNA levels. The relative gene expression was calculated by 2^−ΔΔCt^ method^63^.

### Image analysis and quantification

The stained sections were captured using a 10× (Plan-APOCHROMAT 10×/0.45) or 20× (Plan-APOCHROMAT 20×/0.8) primary objective on a VSlide slide-scanning microscope (Metasystems, Altlußheim, Germany) equipped with filter sets for DAPI (EX350/50 - EM470/40), FITC (EX493/16 - EM527/30) and Cy5 (EX630/20 - 647/long pass). Individual field-of-view images were stitched to produce images of entire DRG and spinal cord sections with microscopic resolution. Images were analyzed with MetaViewer software (Metasystems) to evaluate possible co-existence through channel operations.

To determine the percentage of FRMD6-IR NPs in DRGs, every 4th or 6th, 12 μm-thick section was selected for counting. All counting was conducted with MetaViewer software (Metasystems) and Image J software (1.48 v, National Institutes of Health, Bethesda, MD). The quantification of percentage of FRMD6^+^ NPs was performed following the same procedure as reported in our previous study^64^. To determine the percentages of co-existence of FRMD6 with three classical neuronal phenotypic markers and galanin, 6 DRGs from three animals were selected and counted as previously described^61^. Double-staining was also performed on sections of spinal cord and examined under an LSM 700 confocal microscope (Zeiss, Oberkochen, Germany) as described in our previous work^65^. The cross-sectional area of FRMD6^+^ NPs was measured using Image J software. Only FRMD6^+^ NPs containing a fraction of the nucleus were included. The size distribution was analyzed in Excel 2010 software (Microsoft, Redmond, WA) followed the criteria: small (<300 μm^2^), medium-sized (300–700 μm^2^) and large (>700 μm^2^)^66^. Intensity of FRMD6-immunoreactivity in DRGs, spinal cord and Western blot was also analyzed with Image J software by drawing the region of interest.

### Statistical analysis

Statistical analysis was performed using GraphPad Prism 6.01 software (GraphPad Software, Inc.). All quantification data were expressed as mean ± SEM. Differences of FRMD6-IR NPs and mean intensity of FRMD6-immunoreactivity in DRGs and spinal cord between contra- and ipsilateral side were evaluated with an F test. Comparisons of phenotypic changes of FRMD6^+^ NPs based on expression pattern or neuronal size in contra- and ipsilateral DRGs were performed by two-way ANOVA. *p* < 0.05 was taken as the criterion for statistical significance. All data for quantification analyses were performed by one experimenter who was blind for the surgery.

## Supplementary information

Supplementary information.

## Data Availability

All data generated from this study has been included into this article (and the Supplementary files). The raw data will be available from the corresponding authors upon reasonable request.
