# Supplementary material for: Expression and regulation of FRMD6 in mouse DRG neurons and spinal cord after nerve injury
Source: Sci Rep. 2020 Feb 5;10:1880. doi: 10.1038/s41598-020-58261-7 (PMC7002571; doi:10.1038/s41598-020-58261-7)
Supplement: Supplementary file 1 — Supplementary information. [file 41598_2020_58261_MOESM1_ESM.docx]

**Expression and regulation of FRMD6 in mouse DRG neurons and spinal cord after nerve injury**

Chuang Lyu^1*^, Gong-Wei Lyu^3^, Jan Mulder^2^, Mathias Uhlén^2,4^, Xue-Hui Cai^1^, Tomas Hökfelt^5^ & Tie-Jun Sten Shi^6^*

**Supplementary Figures and Legends**


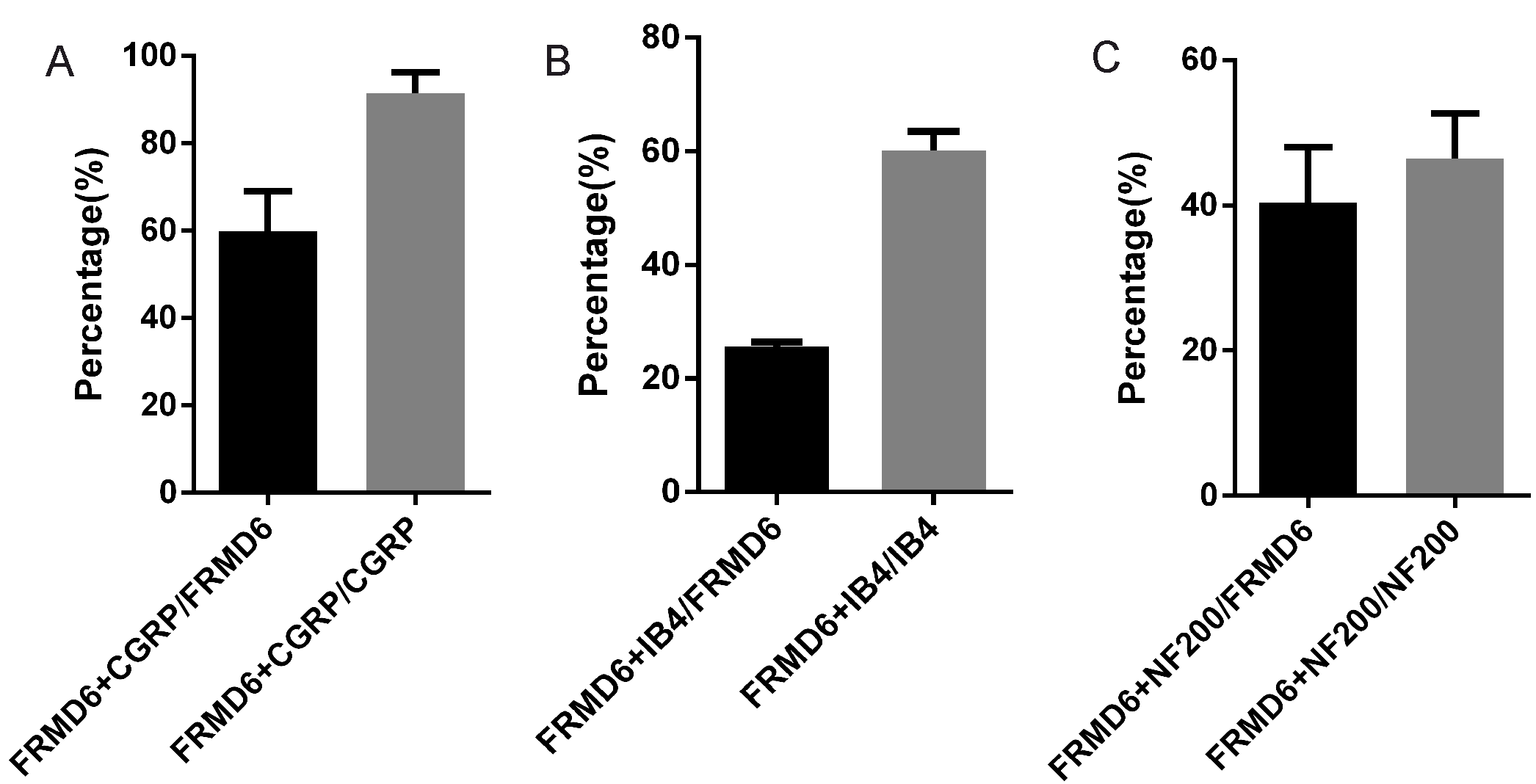


**Figure S1.** FRMD6 co-exists with three neuronal markers in ipsilateral DRGs 1 week after axotomy. Quantification shows the percentage of co-existing NPs in (A) FRMD6^+^ or CGRP^+^ neuron populations, (B) in FRMD6^+^ or IB4^+^ neuron populations and (C) in FRMD6^+^ or NF200^+^ neuron populations.


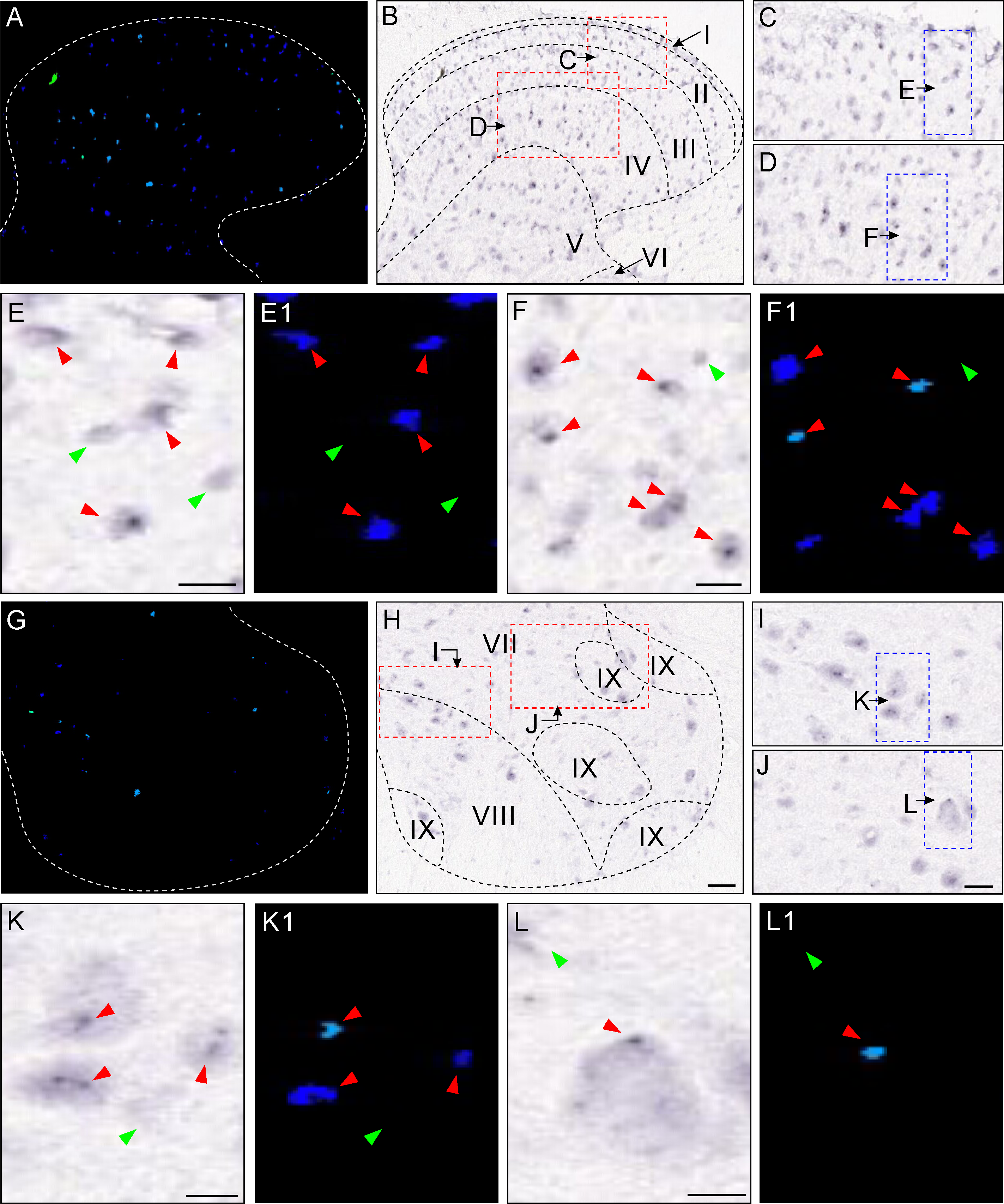


**Figure S2.** FRMD6 mRNA detected by in situ hybridization in counter stained, control mouse spinal cord. (A, E1, F1, G, K1, L1) Dark field micrographs show distribution of FRMD6 mRNA in the dorsal horn (DH) (A, E1, F1) and ventral horn (VH) (G, K1, L1). (B, C, D, E, F) and (H, J, K, L, H) Bright field micrographs with counter staining show distribution of FRMD6 mRNA in DH and VH, respectively. In (B) and (H) nine of the spinal cord layers are indicated by dashed lines. (A-L1) Note numerous FRMD6 mRNA^+^ neurons in all layers. (E-F1, K-L1) High magnification images show FRMD6 mRNA-positive (red arrowheads) and negative (green arrowheads) signals. Note strictly nuclear localization of the transcript. Reproduced from the Allen Brain Atlas ^[41](#_ENREF_41)^. Scale bars indicate 50 μm (A-B, G-H), 25 μm (C-D, I-J) and 10 μm (E-F1, K-F1).


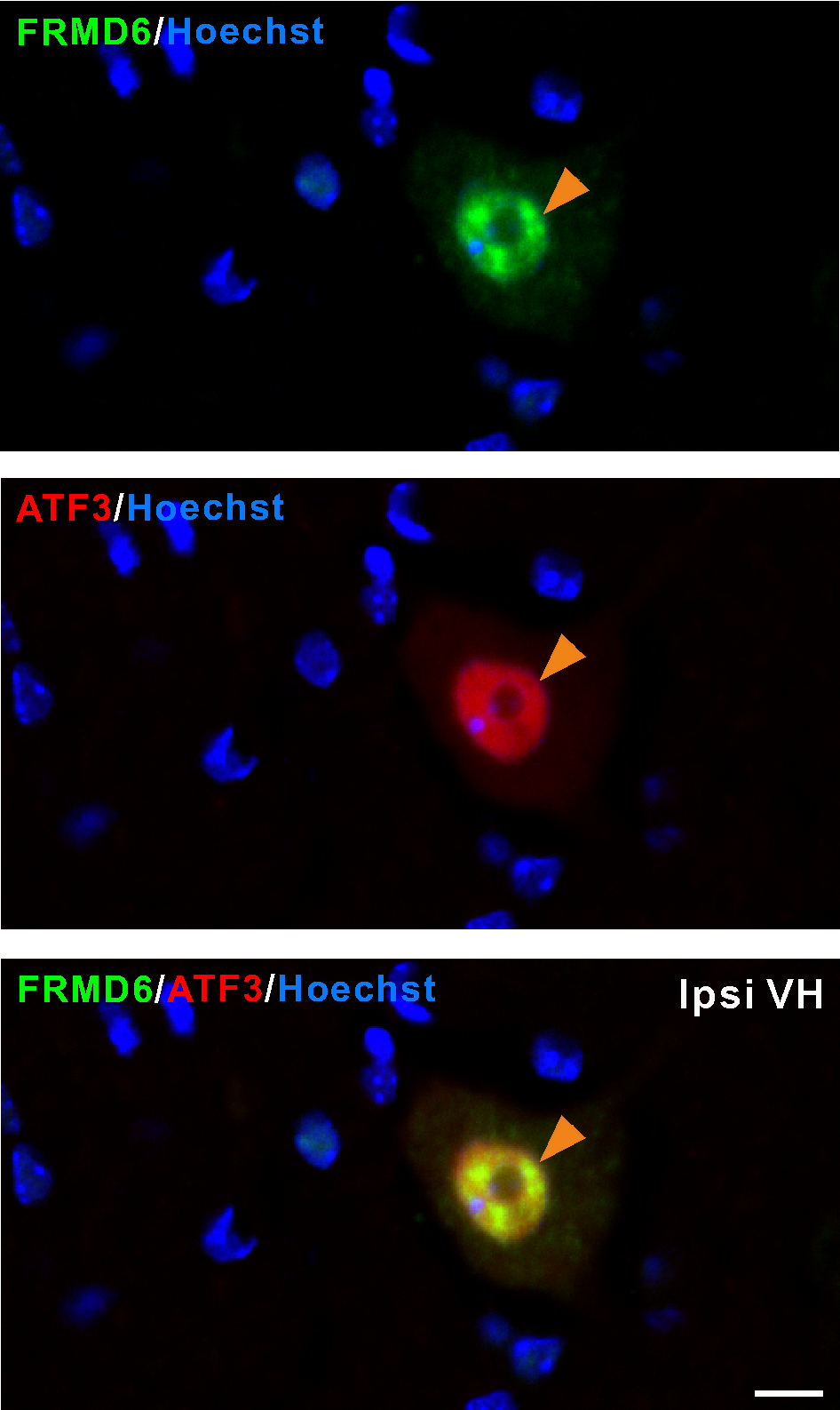


**Figure S3.** Co-localization of FRMD6-immunoreactivity and ATF3-immunoreactivity in the nucleus of motor neurons in ipsilateral ventral horn 7 days after sciatic nerve axotomy. Scale bar indicates 20 μm.


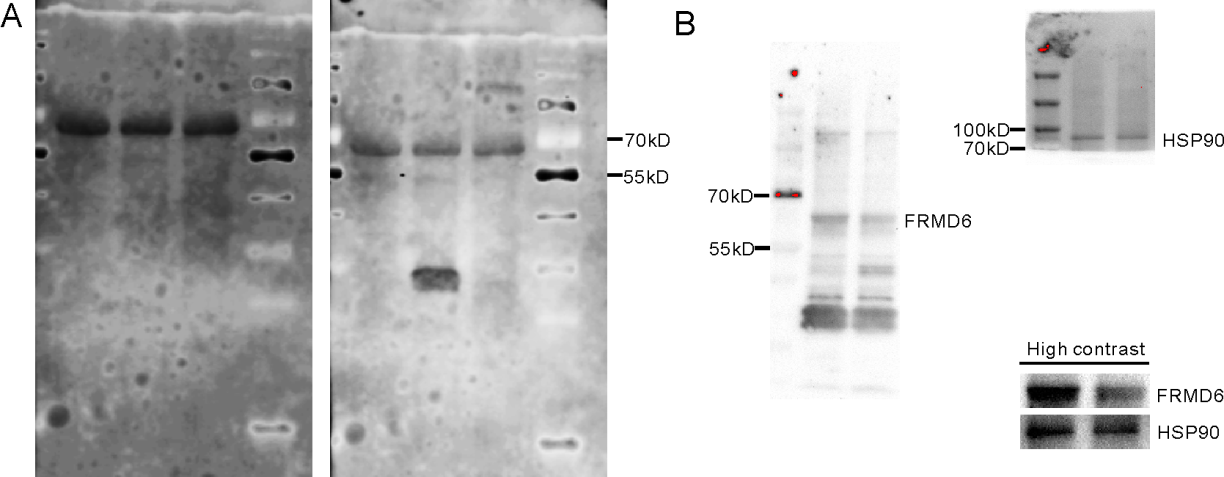


**Figure S4.** The intact immunoblotting membranes for Western blot in this study. (A) The intact WB membrane for Fig. 1A. (B) The intact membrane for Fig. 3D.
